# Feasibility of hemoperfusion using extracorporeal therapy in the horse

**DOI:** 10.3389/fvets.2024.1414426

**Published:** 2024-05-14

**Authors:** Kallie J. Hobbs, Andre N. V. Le Sueur, Megan J. Burke, Bethanie L. Cooper, M. Katie Sheats, Yu Ueda

**Affiliations:** Department of Clinical Sciences, College of Veterinary Medicine, North Carolina State University, Raleigh, NC, United States

**Keywords:** hemoperfusion, equine, extracorporeal therapy, sepsis, cytokines

## Abstract

**Objective:**

Develop, implement, and monitor for adverse effects of, a novel hemoperfusion therapy in adult horses.

**Methods:**

A prospective, observational feasibility study using three healthy adult horses from the North Carolina State University teaching herd. Health status was determined by physical exam, complete blood count, coagulation panel, and serum biochemistry. Each horse was instrumented with a 14 Fr × 25 cm double-lumen temporary hemodialysis catheter and underwent a 240 min polymer-based hemoperfusion session. Horses were administered unfractionated heparin to maintain anti-coagulation during the session. Given the novelty of this therapy in horses, each horse was treated as a learning opportunity that informed an iterative process of protocol development and modification.

**Measurements and main results:**

Our long-term goal is to investigate potential clinical applications of hemoperfusion in horses, including cytokine reduction in horses with severe SIRS/sepsis. Horses were monitored for changes in clinical exam, biochemistry and hematology parameters. Additionally, cytokines were quantified to determine whether extracorporeal hemadsorption therapy alone caused an inflammatory response. Our results show that hemoperfusion therapy was associated with decreased platelet counts and serum albumin concentration. There was no significant change in plasma cytokine concentrations with hemoperfusion therapy. In one horse, the cytokine concentrations decreased, as previously reported with hemoperfusion therapy in humans.

**Hypothesis:**

We hypothesized that hemoperfusion therapy could be performed in healthy adult horses without significant adverse effects.

**Conclusion:**

Polymer-based hemoperfusion is a feasible extracorporeal therapy (ECT) modality for adult horses. Additional studies are needed to further establish clinical protocols, as well as establish efficacy of polymer-based hemoperfusion for treatment of various conditions in horses, including intoxications, immune-mediated conditions, and sepsis.

## 1 Introduction

Hemoperfusion is an extracorporeal blood purification modality that consists of mass separation and absorption by a solid agent (sorbent) (1). Sorbents have been studied for many years, and charcoal and resins are hemoperfusion’s most studied sorbent surface (1, 2). While early hemoperfusion techniques led to adverse reactions and had issues with safe storage and priming, recent biocompatible sorbent materials have been safely used for hemoperfusion in various clinical settings (3).

For use in humans, Cytosorb^®^ is a novel non-pyrogenic, sterile, single-use, polymer-based column that contains adsorbent polymer beads designed to remove solutes less than 60 kD, and has shown promising cytokine removal in patients with severe septic shock (4). Hemoperfusion has been widely used in human medicine for treating toxin ingestion (5) and sepsis (6). In septic patients, there is improvement in immune cell function characterized by reduced neutrophil extracellular trap formation and improved leukocyte response to antigen stimulation (7). Hemoperfusion has also been used in small animal veterinary patients for treatment of exogenous and endogenous intoxications, immune-mediated conditions (8), and heatstroke with cytokine dysregulation (9).

While there are reports in the literature on hemodialysis and plasmapheresis in horses, the use of hemoperfusion in large animals has yet to be reported (10). Conditions treated by hemoperfusion in other species are primarily treated with supportive care in horses. A recent publication by Hobbs et al. shows that a polymer-based column known as VetResQ^®^, which uses the same technology as CytoSorb^®^, can filter cytokines out of equine blood *ex vivo*. This product also demonstrated high removal of non-steroidal anti-inflammatory drugs and aminoglycosides from equine blood *ex vivo* (11).

To date, no studies have reported use of hemoperfusion in the horse. Therefore, the primary objective of this study was to investigate the feasibility of hemoperfusion in adult horses by conducting a prospective, observational feasibility study in three healthy adult horses. Safety of treatment was investigated through measurement of plasma cytokine concentrations, hematology and biochemistry parameters, and physical exam monitoring, before, during and after treatment. Our hypothesis was that hemoperfusion therapy could be performed safely in healthy adult horses without significant adverse effects.

## 2 Methods

### 2.1 Animals and experimental design

Three healthy adult horses ranging from 440 to 510 kg from the North Carolina State University Teaching herd were used for this feasibility study. An initial list of eligible horses was based on adult age, bilateral patent jugular veins, healthy, temperament suitable for standing handheld or tied in a stall, and availability. Beyond that, horses were chosen randomly. Health status was determined by normal hematology and biochemistry results, as well as a normal temperature, pulse and respiration. This study was approved by the NC State Institutional Animal Care and Use Committee (IACUC 23-110).

### 2.2 Perfusion pump and column

Hemoperfusion was performed with a VetSmart machine (Medica-Spa, Medolla, Italy), and a VetResQ^®^, Cytokine, and Lipophilic Drug Removal – 300 mL device (CytoSorbents Inc., Monmouth Junction, NJ). The columns were primed with sterile 0.9% NaCl saline (2 L total) before therapy. In this study, the average blood flow rate achieved was 150 mL/min (range of 80–200 mL/min). This was the average rate that maintained arterial and venous chamber pressures within the range of −200 to +200 mm Hg.

### 2.3 Catheters

**Figure 1:**
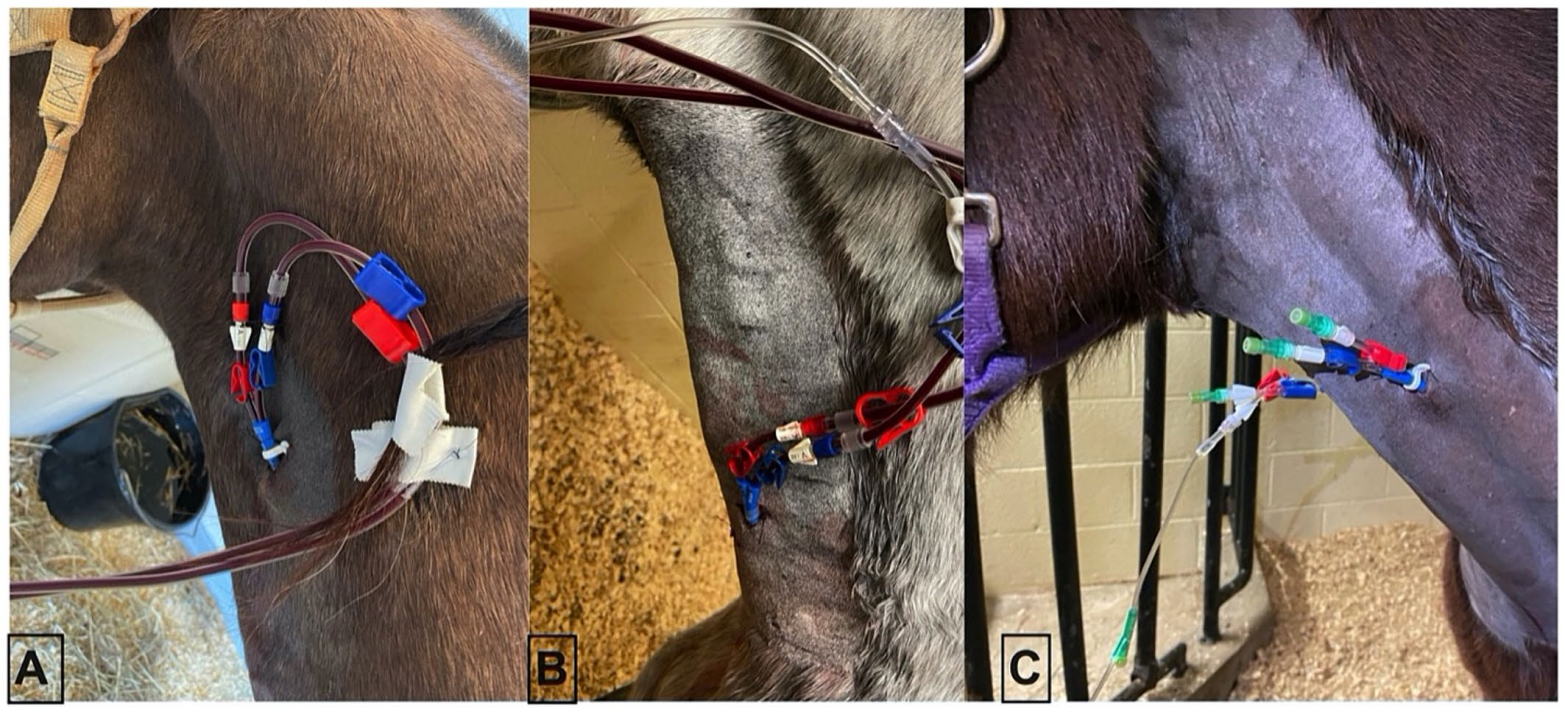
Three catheter placement locations in an adult horse. **(A)** Proximal placement, **(B)** distal placement, and **(C)** mid-neck placement.

All horses had a 14 Fr × 25 cm double-lumen temporary hemodialysis catheter (Arrow, Morrisville, NC) placed sterilely in the left or right external jugular vein without complication using the modified Seldinger technique. One horse was administered 0.01 mg/kg of detomidine before placement. Catheters were placed in three different locations in each of the horses (top 1/3 of the neck, middle 1/3 of the neck, and thoracic inlet) in order to inform subjective assessment of impact of catheter location on hemoperfusion (Figure 1).

### 2.4 Activated clotting time measurement, coagulation measurement, and heparinization

Coagulation panel measurements included activated partial thromboplastin time (aPTT), prothrombin time (PT), fibrinogen, and international normalized ratio (INR) (North Carolina State Clinical Pathology), and an activated clotting time (ACT) using a Medtronic ACT 2 Plus. ACT was measured every 15–45 min throughout the treatment, and a coagulation panel was measured at baseline, post-filtration, and 24 h. For Horse 1, an initial bolus of unfractionated heparin (UFH) of 30 UI/Kg was administered for a target ACT of 300–400 s. This target was 2 to 2.5x published baseline values in horses (12), which is common practice for ECT in human patients (13, 14). While filtering Horse 1 we determined that cell aggregation were obstructing the cartridge. This was determined through a series of troubleshooting steps that evaluated line patency, catheter positionality and the effects of column replacement. To avoid the clogging of the column with cell aggregation, we increased the target ACT to 700–800 s and we saw significantly improved blood flow. Subsequently, we increased the initial UFH bolus to 50 UI/kg for Horse 2, and 60 UI/kg for Horse 3. The adjustment in target ACT to 700–800 s significantly improved blood flow without complications. Low doses of UFH were supplemented via CRI within the (ECT) circuit (0–50 UI/Kg/h, IV) to maintain target ACT.

### 2.5 Hematology and biochemical parameters

Biochemistry (Stago Chemistry Analyzer, Mount Olive, NJ) and hematology (Avida 1,000 Hematology, Siemens Healthcare, Erlangen, Germany) analysis was performed prior to, and immediately and 24 h post-filtration. Analyzers were located on site.

### 2.6 Clinical exam parameters

Clinical exam parameters including attitude, rectal temperature, respiratory, and heart rate were recorded before treatment started, at two-hours treatment, at the end of treatment, and 24 h post-treatment.

### 2.7 Cytokine measurement

For cytokine measurement, anticoagulated blood was collected at 0,15,30,45,60,90,120,180, and 240 min. Measurement of cytokines interleukin-1beta (IL-1β), interleukin 5 (IL-5), interleukin 6 (IL-6), interferon-gamma (IFN-γ), interleukin 8 (IL-8), interleukin 10 (IL-10), and tumor necrosis factor alpha (TNF-α) was performed using an equine-specific Milliplex^®^ Map Magnetic Bead Panel (EMD Millipore, Billerica, MA, United States) per the manufacturer’s protocol using a previously validated method (15). Samples were analyzed in duplicate. Briefly, for the assay, 96-well assay plates were washed using kit wash buffer before use. Background (assay buffer), standard, and control wells were loaded onto the plate. Next, 25 μL of each sample and assay buffer were plated in sample wells, and each sample was plated in duplicate. Plates were covered and incubated on an orbital shaker overnight at 4°C in the presence of magnetic beads coated with fluorescently labeled capture antibodies for each analyte. Beads were then washed and incubated with biotinylated detection antibodies, followed by the addition of streptavidin phycoerythrin. Beads were rewashed before resuspension in 150 μL of sheath fluid, and sample analysis using a minimum count of 50 beads per well was used for inclusion in the analysis. The mean of the duplicate samples was used for analysis. The plate was run on a Bio-Rad Luminex Bio-Plex 200 Suspension Array Stem (Luminex, Austin, TX), and Bioplex Manager Software Version 6.2 was used for analysis.

### 2.8 Statistics

Due to the small number of animals in this study, data were not tested for normality. Median and range was determined for physical exam, hematology and biochemistry parameters before filtration, after filtration and 24 h post filtration. No statistical analysis of cytokines was performed and data is reported for each measured time point.

## 3 Results

All horses completed 240 min of hemoperfusion treatment without serious adverse event (i.e., jugular vein thrombosis, altered cardiovascular status, abnormal bleeding, colic, behavioral noncompliance). Catheter placement was well tolerated in all locations and did not affect overall blood volume circulated over the 240 min (average 150 mL/min = 1.5x blood volume). For all catheter placements, a small stab incision was necessary due to the large diameter of the catheter. Subjectively, catheter placement in the proximal 1/3 of the neck was more sensitive to occlusion when the horse was pulling hay from the hay bag or moving in the stall. Catheters were left in place until ACT values returned to baseline values.

Baseline ACT measurements for the three horses were 133, 193 and 137 s, consistent with previously published reference ranges of 120–180 s (12, 16). ACT values between 700 and 824 s were necessary to achieve consistent flow that maintained appropriate venous and arterial pressures of −200 to +200 mm Hg across the hemoperfusion pump and column. For two of the horses, a 30 IU/Kg bolus of UFH resulted in ACT values of 328 and 342 s, respectively. Horse 2 received an additional 20 IU/Kg bolus to reach the target starting ACT of 700–800 s. For the third horse, a 60 IU/Kg bolus of UFH resulted in an ACT of 705 s. During treatment, UFH was given as a continuous rate infusion (CRI) from 0 UI/Kg/h to 50 UI/Kg/h to maintain appropriate anticoagulation. PT, fibrinogen and INR values remained consistent before and after treatment. Activated partial thromboplastin time (aPTT) was increased to greater than >120 in all three horses at the end of hemoperfusion but returned to within reference ranges (37.3–51.4 s) at the 24 h time point (Supplementary Table S1A).

Complete blood cell counts and biochemistry parameters remained within published reference ranges (17). While absolute counts were within accepted reference ranges, there was a decrease in numbers of neutrophils (median before 3.77 ×10^3^ uL range 2.25–5.88, median after 2.19 range 2.11–5.12) and platelets (median before 130 ×10^3^ uL range 132–140, median after 93 range 80–143). Neutrophil numbers returned to baseline values by 24 h post-treatment. We also observed a moderate decrease in total calcium (*n* = 3), albumin (*n* = 3), and potassium (*n* = 2) (Supplementary Table S1B). Physical exam parameters remained within normal reference ranges throughout treatment and horses stood comfortably and ate hay from a haynet throughout hemoperfusion therapy (Table 1). One horse did experience a mild nosebleed several hours after therapy. Upon further investigation it was discovered that the horse had been used for a nasogastric teaching lab several days prior. It is possible that anticoagulation in this horse caused bleeding from recent trauma to the ethmoid turbinates. The bleeding was so transient that no additional diagnostics were performed.

**Table 1:** Physical exam parameters from three horses at baseline, 2 h, and 4 h of hemoperfusion therapy followed by a 24 h post hemoperfusion exam.

Horse	Temp-Fahrenheit/Celsius	HR- beats per minute	Resp-breaths per minute	Mucus membranes-MM	Attitude
Horse 1 – T0	99.2/37.33	36	18	Moist/Pink	BAR
Horse 1 – T120 min	98.7/37.05	36	18	Moist/Pink	BAR
Horse 1 – T240 min	98.7/37.05	36	18	Moist/Pink	BAR
Horse 1 – 24 h Post	99.1/37.27	36	12	Moist/Pink	BAR
Horse 2 – T0	98.9/37.16	36	16	Moist/Pink	BAR
Horse 2 – T120 min	99.1/27.27	36	16	Moist/Pink	BAR
Horse 2 – T240 min	98.9/37.16	26	14	Moist/Pink	BAR
Horse 2 – 24 h Post	99/37.22	32	12	Moist/Pink	BAR
Horse 3 – T0	99.9/37.72	30	18	Moist/Pink	BAR
Horse 3 – T120 min	99.3/37.38	30	18	Moist/Pink	BAR
Horse 3 – T240 min	99.6/37.56	30	18	Moist/Pink	BAR
Horse 3 – 24 h Post	99.3/37.38	32	14	Moist/Pink	BAR

**Figure 2:**
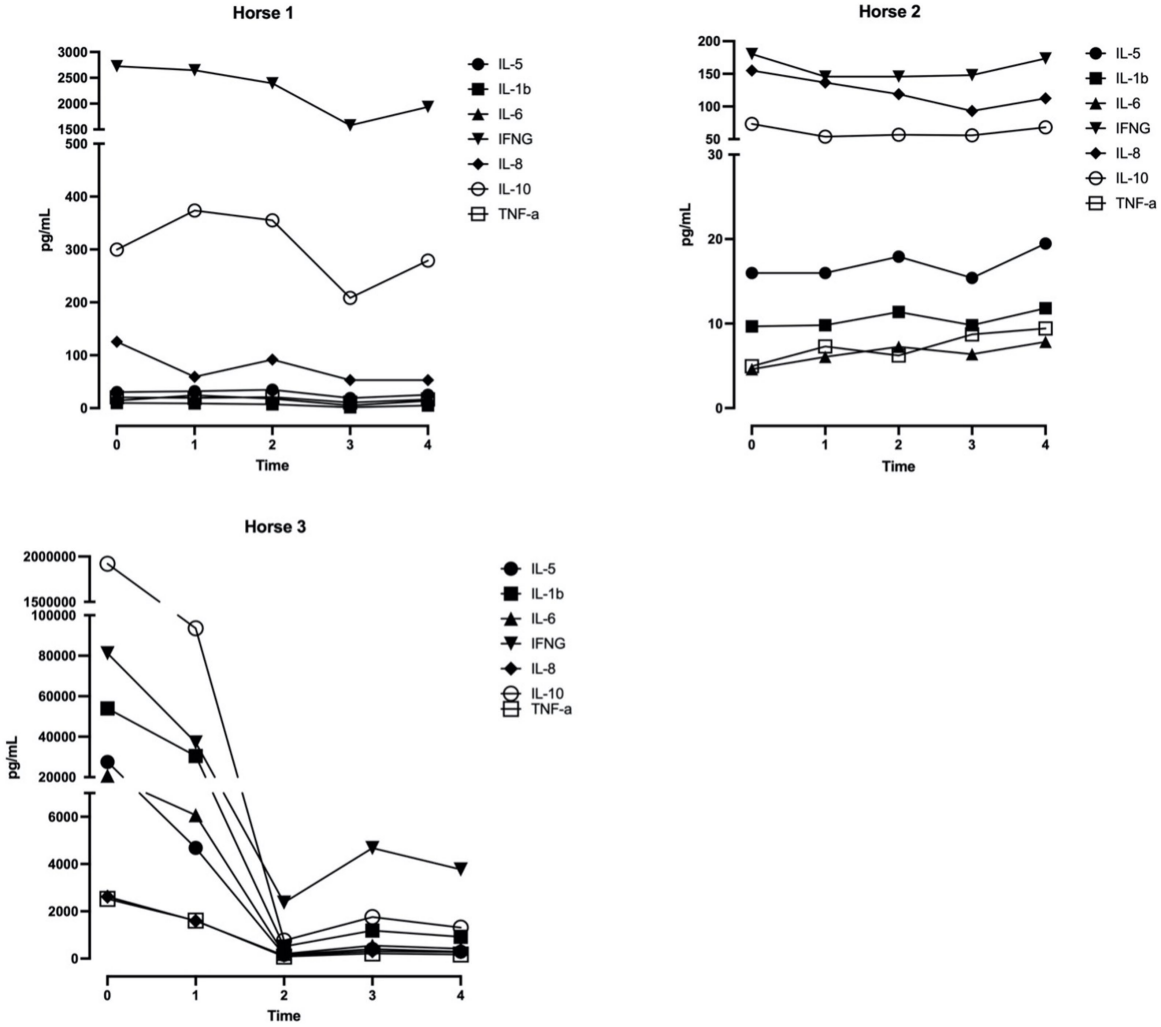
Concentrations of IL-5, IL-1β, IL-6, IFN-g, IL-8, IL-10, TNF-α over 4 h of filtration with a cytokine adsorption device (VetResQ; CytoSorbents Corp) in plasma obtained from heparinized whole blood samples, each collected from a healthy adult horse (*n* = 3). For each time point, each shape represents a single cytokine over 4 h. Lines connect results for same horses.

Levels of IL-1β, IL-5, IL-8, IL-10, and TNFα remained consistent in two horses from baseline through the duration of therapy. In one horse (that experienced hives before initiation of therapy), levels of IL-1β, IL-5, IL-8, IL-10, and TNFα were much higher at baseline than the other two horses. Interestingly, cytokine levels in this horse decreased rapidly during the first 2 hours of filtration and then normalized during the final 2 hours of filtration (Figure 2).

## 4 Discussion

For this study, we hypothesized that hemoperfusion therapy could be performed in healthy adult horses without significant adverse effects. To our knowledge, this is the first study to describe the use of extracorporeal hemoperfusion therapy in horses *in vivo*. The findings of this prospective, observational feasibility study support hemoperfusion as a feasible and safe treatment modality in adult horses, and additional studies on potential clinical applications of this treatment modality should be considered.

All horses received 240 min extracorporeal hemoperfusion therapy without any significant adverse effects detected. However, it is important to note that we were only able to achieve blood flow rates of 150 mL/min which correlates to 1.5x blood volume for the horses selected for this study. Generally, hemoperfusion targets a 5x volume turnover; but there is currently no evidence for a target filtration blood volume that would impact clinical outcomes in horses with different problems or conditions (18). We did observe a rapid decrease in cytokines during the first 2 hours of filtration in one horse, so it is possible that filtration of even a small blood volume could impact cytokine levels. It is also possible that this horse would have experienced rapid cytokine decrease even without filtration. Additional controlled studies will be needed to better determine the impact of blood filtration volumes on changes in levels of systemic cytokines.

In other species, such as dogs and humans, 2.5x baseline ACT is typically required to facilitate successful blood circulation and avoid clotting in the extracorporeal circuit (13). For the horses in our study, approximately 3–4x baseline ACT was required to achieve a consistent flow through the circuit. Previous studies have shown several differences between equine platelets and platelets of other species, including response to anticoagulants (19, 20). In these studies it was elucidated that equine platelets experience rouleau formation not found in other species as well as heparin dependent clumping of platelets (21). It is possible that these differences explain the higher level of heparinization required for hemoperfusion in horses. Prothrombin time (PT) and international normalized ratio (INR) remained within the reference interval (10.3–12.9 s). Activated partial thromboplastin time (aPTT) increased as expected with anticoagulation therapy by heparin. Interestingly even the initial bolus of heparin resulted in aPTT higher than the laboratory measurable range (0–120 s) suggesting it may not be an ideal parameter for heparin monitoring in the horse. All horses returned to baseline level of ACT within 6 h after treatment discontinuation, at which time the catheters were removed.

In other species, ECT associated changes in hematology and biochemistry parameters include decreases in platelets, albumin and total calcium (22, 23). Our previously published *ex vivo* study and current feasibility study data show similar findings (11). None of these parameters fell below clinically accepted reference ranges with the 240 min treatment (Table 1). This suggests that horses with protein-losing conditions may require additional monitoring for changes in electrolytes and serum albumin concentrations during hemoperfusion, and may require additional colloid and/or electrolyte supplementation.

In people undergoing Cytosorb treatment for sepsis, the columns are exchanged every 12 to 24 h. For treatment of intoxications, the manufacturer recommends exchange of columns hourly (24–26). In Horse 1, the column was exchanged at 30 min, 60 min, and 120 min. This was in response to high transmembrane pressure alerts presented by the ECT machine, which we ultimately attribute to clogging with cell aggregation in the filter since these alerts ceased once ACT was increased above 700 s (27). After filtering Horse 1, we adjusted our target ACT from 350 s (approximately 2.5x baseline) to 700–800 s (approximately 5x baseline). In Horses 2 and 3, one column was used for the entire the 240 min hemoperfusion session (Supplementary Table S2), which lends support to our decision to increase target ACT. While no adverse effects were detected when one column was used for the full 240 min, we have yet to determine how quickly a column will reach saturation for various disease states in the horse.

In our study, Horse 2 maintained consistent cytokine levels at all sampled time points. Horse 1 had higher IL8 and IFN-γ at baseline compared to horse 2. While Horse 1 was considered healthy enough for participation in this research study, this 25 year old was previously diagnosed with Pituitary Pars Intermedia Dysfunction (PPID) and on daily Prascend treatment. A previous study by MacFarlane et al. shows that healthy older horses (>16 years) have increased relative mRNA expression of several cytokines including interleukin (IL)-6, IL-8, and interferon-γ in white blood cells (WBCs) (28). Previous studies also show that horses with PPID have increased relative mRNA expression of IL8 in WBCs (28, 29). Therefore, cytokine differences in Horse 1 were likely the result of advanced age, PPID status, or both. Of potential relevance to our investigation, both IL8 and interferon-γ decreased over the course of hemoperfusion therapy in Horse 1. Horse 3 had markedly elevated baseline cytokines, including IL6, IL5, IL1b, and IL10. This was surprising, given that HR, RR, rectal temperature, and hematology and biochemical parameters were normal at the start of the study, and this horse was not predisposed to elevated cytokines due to advanced age or chronic health conditions. In hindsight, it was noted on physical exam that Horse 3 had urticaria the morning of the study, but it was not deemed concerning enough to remove the horse from study participation. A previous study by Hinden et al. shows elevated cytokine mRNA expression, including IL4, *L-13*, *TSLP*, and *IL-4Rα*, in lesional skin in horses with recurrent urticaria, but this study did not investigate changes in systemic cytokines (30). Despite this, we suspect the cytokine elevation in Horse 3 was likely associated with urticaria. Of potential relevance to our investigation, all elevated cytokines decreased over the course of hemoperfusion therapy in Horse 3. While the goal of this observational study was to investigate feasibility and safety, rather than efficacy, the decrease in systemic cytokines seen with Horse 1 and Horse 3 does provide proof of concept data for polymer-based hemoperfusion as a gradient dependent cytokine removal method in horses. It is also important to note that hemoperfusion with this column did not remove cytokines completely, which is potentially advantageous since complete removal could be detrimental to immune cell function (31).

This study had several limitations, with the primary limitation being small sample size. A larger sample size would have allowed for continued optimization of catheter placement and flow rate. Another limitations was that the filtration time and blood volume filtered were less than typical targets for human and small animal patients, which could underrepresent the potential for complications, and hematologic and biochemical derangements. At the time of this study, only pediatric bloodlines were available (85 mL capacity). Since the completion of this study, the authors have been able to obtain an average of 250 mL/min of blood flow using large bore hemoperfusion lines (190 mL capacity) (Medica M90052, Medolla, Italy), paired with a catheter setup of 14 Fr × 15 cm double-lumen in the proximal aspect of the vein and 10 Fr × 15 cm single lumen in the distal aspect of the vein. Additionally, while evidence for safety of this therapy is supported by the maintenance of normal physical exam, hematologic and biochemical profiles, and cytokines that decreased instead of increasing, these were healthy animal. Additional research regarding safety of hemoperfusion in sick horses will be needed. We also acknowledge that because this was a feasibility study in which we were making frequent protocol modifications, there wasn’t one tested protocol. We are planning additional research with appropriately sized blood lines and a pump calibrated for use in horses in order to validate a protocol and share detailed data (e.g., blood flow rate, access pressure, return pressure, transmembrane pressure). Lastly, we only tested one polymer-based hemoperfusion column. There are several other types of columns on the veterinary market that either work through a carbon-based mechanism or selective filter-based mechanism. While several of these column types have been used in small animal veterinary patients, there is currently no data on their safety or efficacy in equine patients. There are also aspects of feasibility that we did not address in this study, including financial costs of this therapy for clients, expense of equipment to veterinary facilities, and the intense level of management these patients require during hemoperfusion. It remains to be seen whether benefits of this therapy for patients will balance the very real costs.

To our knowledge, this is the first report of hemoperfusion extracorporeal therapy in adult horses *in vivo*. The results of this study show that polymer-based hemoperfusion ECT is both feasible and safe in adult horses. We also show proof of concept data supporting further investigation of polymer-based hemoperfusion as a method for systemic cytokine removal in horses. Additional studies are needed to establish efficacy of polymer-based hemoperfusion for treatment of various conditions in horses, including intoxications, immune-mediated conditions, and sepsis.

## Data availability statement

The original contributions presented in the study are included in the article/Supplementary material, further inquiries can be directed to the corresponding authors.

## Ethics statement

The animal study was approved by North Carolina State University-IACUC. The study was conducted in accordance with the local legislation and institutional requirements.

## Author contributions

KH: Data curation, Formal analysis, Investigation, Writing – original draft, Writing – review & editing. AL: Data curation, Investigation, Project administration, Writing – original draft, Writing – review & editing. MB: Data curation, Investigation, Project administration, Writing – review & editing. BC: Data curation, Investigation, Writing – review & editing. MS: Funding acquisition, Investigation, Resources, Writing – original draft, Writing – review & editing. YU: Funding acquisition, Investigation, Project administration, Writing – review & editing.
