# Supplementary material for: Feasibility of hemoperfusion using extracorporeal therapy in the horse
Source: Front Vet Sci. 2024 May 14;11:1414426. doi: 10.3389/fvets.2024.1414426 (PMC11128652; doi:10.3389/fvets.2024.1414426)
Supplement: Supplementary file 1 [file Table_1.DOCX]

| **Horse** | **WBC** | **Neutrophils** | **Lymphocytes** | **Monocytes** | **Eosinophils** | **Basophils** | **RBC** | **Hemoglobin** | **Hematocrit** | **Platelets** | **Fibrinogen** | **PCV** | **PT** | **apTT** | **INR** |
| --- | --- | --- | --- | --- | --- | --- | --- | --- | --- | --- | --- | --- | --- | --- | --- |
| Horse 1 Base | 6.68 | 3.741 | 2.405 | 0.134 | 0.401 | 0 | 6.59 | 11.7 | 32.2 | 140 | 300 | 30 | 12.5 | 35.5 | 1.1 |
| Horse 1 -end treatment | 4.7 | 2.11 | 1.92 | 0.17 | 0.44 | 0.03 | 5.19 | 9 | 25.1 | 93 |  |  | 12.9 | >120 | 1.15 |
| Horse 1-24hr Post | 6.23 | 3.987 | 1.744 | 0.125 | 0.374 | 0 | 6.15 | 10.7 | 29.7 | 84 | 251 | 29 | 12.7 | 37.4 | 1.12 |
| Horse 2 Base | 7.15 | 2.25 | 4.219 | 0.143 | 0.21 | 9 | 6.78 | 12 | 33 | 132 | 100 | 33 | 11.7 | 44.2 | 1.01 |
| Horse 2 -end treatment | 6.26 | 2.19 | 3.6 | 0.188 | 0.188 | N/A | 6.24 | 10.8 | 31.6 | 80 | 200 | 29 | 12.1 | >120 | 1.1 |
| Horse 2-24hr Post | 6.21 | 3.44 | 2.14 | 0.2 | 0.3 | 0.01 | 6.97 | 13.2 | 36.6 | 141 | 400 | 36 | 12.2 | 45.5 | 1.07 |
| Horse 3 Base | 9.2 | 5.88 | 2.7 | 0.36 | 0.368 | 0.092 | 7.01 | 13.2 | 37.4 | 138 | 300 | 36 | 12.2 | 47.5 | 1.07 |
| Horse 3 -end treatment | 5.19 | 5.12 | 1.166 | 3.4 | 0.052 | N/A | 5.12 | 9.8 | 27.8 | 142 | 200 | 27 | 12.2 | >120 | 1.2 |
| Horse 3-24hr Post | 8.56 | 3.5 | 4.1 | 0.2 | 0.48 | 0 | 8.18 | 14.3 | 40.7 | 86 | 300 | 37 | 12.1 | 48.2 | 1.06 |

**Supplemental Table 1A**: Complete blood cell count and coagulation analysis from 3 horses pre filtration with VETRESQ® and after 4 hours of filtration on an extracorporeal therapy circuit. WBC=white blood cell count, RBC=red blood cells, PCV= packed cell volume, PT= prothrombin time, apTT= activated partial thromboplastin time, INR=international normalized ratio.

| Horse | Glucose | Urea | Creatine | Phos | Calcium | Mg | Total Protein | Albumin | Globulin | Ratio | Trig | Bili | Alk | AST | GGT | SDH | CK | Sodium | Potassium | Chloride | Bicarb |
| --- | --- | --- | --- | --- | --- | --- | --- | --- | --- | --- | --- | --- | --- | --- | --- | --- | --- | --- | --- | --- | --- |
| Horse 1 Base | 89 | 17 | 1.4 | 3 | 13 | 1.8 | 7.3 | 3.1 | 4.2 | 0.74 | 35 | 1.1 | 262 | 285 | 14 | 1.5 | 337 | 134 | 4.6 | 99 | 25 |
| Horse 1 -end treatment | 90 | 1 | 1.4 | 3.1 | 11.1 | 1.9 | 6.6 | 2.6 | 4 | 0.65 | 26 | 0.9 | 243 | 242 | 12 | <0.5 | 307 | 140 | 2.9 | 102 | 28 |
| Horse 1-24hr Post | 83 | 16 | 1.5 | 3.4 | 12.1 | 1.5 | 6.8 | 2.8 | 4 | 0.7 | 26 | 1.1 | 236 | 245 | 12 | <0.5 | 325 | 134 | 5.6 | 100 | 28 |
| Horse 2 Base | 89 | 14 | 1.6 | 2.6 | 11.8 | 1.6 | 7.2 | 3 | 4.2 | 0.71 | 59 | 0.8 | 195 | 212 | 12 | 0.5 | 189 | 134 | 3 | 98 | 24 |
| Horse 2 -end treatment | 93 | 16 | 1.1 | 4 | 10.9 | 2 | 6 | 2.7 | 3.3 | 0.82 | 27 | 0.9 | 275 | 251 | 11 | 0.5 | 232 | 142 | 4.4 | 110 | 25 |
| Horse 2-24hr Post | 87 | 16 | 1.2 | 3.9 | 11.9 | 1.9 | 6.6 | 2.7 | 3.9 | 0.69 | 39 | 1.4 | 296 | 279 | 14 | 0.5 | 689 | 135 | 4.1 | 100 | 25 |
| Horse 3 Base | 94 | 17 | 1.6 | 2.2 | 12 | 1.8 | 7 | 3.1 | 3.9 | 0.79 | 37 | 0.6 | 185 | 212 | 9 | 0.5 | 183 | 133 | 4.3 | 96 | 26 |
| Horse 3 -end treatment | 59 | 12 | 1 | 1.6 | 8 | 1.3 | 4.7 | 2 | 2.7 | 0.74 | 21 | 0.4 | 137 | 137 | 6 | 0.5 | 123 | 142 | 2.6 | 116 | 17 |
| Horse 3-24hr Post | 89 | 14 | 1.6 | 2.6 | 11.8 | 1.6 | 7.2 | 3 | 4.2 | 0.71 | 59 | 0.8 | 195 | 212 | 12 | 0.5 | 189 | 134 | 3 | 98 | 24 |

**Supplemental table 1B**: Complete biochemistry parameters from 3 horses pre filtration with VETRESQ® and after 4 hours of filtration on an extracorporeal therapy circuit. Base = baseline, 4H = after 4 hours Phos=phosphorus, Ca=calcium, Mg=magnesium, Trig=triglycerides, Bili=total bilirubin, ALK=alkaline phosphatase, AST=aspartate transaminase, GGT=gamma-glutamyltransferase, SDH=sorbitol dehydrogenase, CK=creatine kinase, Na+=sodium, K+=potassium, Cl-=chloride, Bicarb=bicarbonate.
